# Chronic fusiform extracranial vertebral artery aneurysm with recurrent posterior circulation emboli: Case report and review of the literature

**DOI:** 10.1177/15910199211018581

**Published:** 2021-05-18

**Authors:** Katherine Evans, Ralf-Björn Lindert, Richard Dyde, George H Tse

**Affiliations:** 1 Academic Department of Neurosciences, Royal Hallamshire Hospital, Sheffield Teaching Hospitals NHS Trust, Sheffield, UK; 2 Department of Neuroradiology, Royal Hallamshire Hospital, Sheffield Teaching Hospitals NHS Trust, Sheffield, UK

**Keywords:** Vertebral artery, aneurysm, embolisation, stroke, dissection, embolism

## Abstract

We report a case of a 64-year-old man with a fusiform right extracranial vertebral artery aneurysm, spanning over half the extra-cranial V2 (foraminal) segment, presenting with recurrent multi-focal posterior circulation embolic ischaemic stroke. The patient was treated with endovascular embolisation of the right vertebral artery to prevent further thrombo-embolic events. Distal and proximal occlusion of the aneurysmal vertebral artery was performed with a micro-vascular plug with partial aneurysm sack embolisation to aid thrombosis and reduce the risk of recanalisation. Two months post procedure MR angiography confirmed successful aneurysm occlusion with no post-procedural complication. The patient returned to his normal independent life. Endovascular treatment with vessel sacrifice is an effective treatment with low morbidity and we believe the MVP device to be a efficacious option in the vertebral artery.

## Introduction

Extracranial vertebral artery aneurysms are uncommon and the vast majority of reports are associated with trauma representing pseudo-aneurysm formation and acute. Reported causes have included such obscure cases as being gored by an ox,^1^ chiropractic manipulation,^2^ snowboarding injuries^3^ and ‘head-banging’ to rock music.^4^ Primary (non acute traumatic) aneurysms are even rarer and arise due to connective tissue diseases; predominantly Ehlers-Danlos syndrome, Marfan syndrome and Neurofibromatosis, and much less commonly secondary to arteriosclerosis.^5^

Extracranial vertebral artery aneurysms can present following rupture and symptoms related to acute haemorrhage,^6–8^ mass effect causing dysphagia,^9^ neck pain^10–15^ and radiculopathy.^14^,^16^,^17^ Presentation also includes posterior circulation ischemia and infarction with clinical features of dizziness, diplopia, nausea, dysarthria, dysphagia, ataxia, unilateral limb weakness and visual field defects. In general posterior circulation strokes account for 20–25% of all ischaemic stroke.^18^

We report an unusual case of a growing chronic long length fusiform vertebral artery aneurysm extending over half the length of the extra-cranial segment presenting with recurrent multi-focal embolic posterior circulation acute infarction which was subsequently treated with endovascular embolisation. In addition we have reviewed the literature on treatment of chronic and perceived primary extracranial vertebral artery aneurysms and excluded acute trauma cases.

## Case report

A 64-year-old man presented with acute onset of dizziness and a left sided field defect. The patient complained of ‘dazzling bright lights’ in the left lower field of vision. He had no headache or neck pain and there was no recent trauma. Past medical history included cholecystectomy and migraine with aura, but no regular medications. Subsequently in the patient’s admission further questioning by the interventional neuroradiologist revealed the patient had fallen down subway stairs twenty years previously with significant trauma but did not receive any treatment. On examination there was a left lower homonymous quadrantinopia. National Institute of Health Stroke Scale of 1 was attributed to the partial visual field defect.

Routine biochemical and hematological investigations were all within normal limits except a mildly elevated cholesterol at 5.1 mmol/L and non-HDL 3.5 mmol/L. ECG demonstrated sinus rhythm. Blood pressure recorded 181/92 mmHg with the remaining observations within normal parameters. The patient was a semi-retired publican, a non-smoker and consumed 20units of alcohol per week. Previous soft tissue neck imaging from 2015 for an intramuscular lipoma identified a right vertebral artery aneurysm with cervical spine remodeling, the patient was asymptomatic and no follow up imaging was arranged. Although dedicated vascular imaging was not undertaken at that time maximal axial aneurysm dimension measured approximately 13 mm maximally.

**Figure 1.**
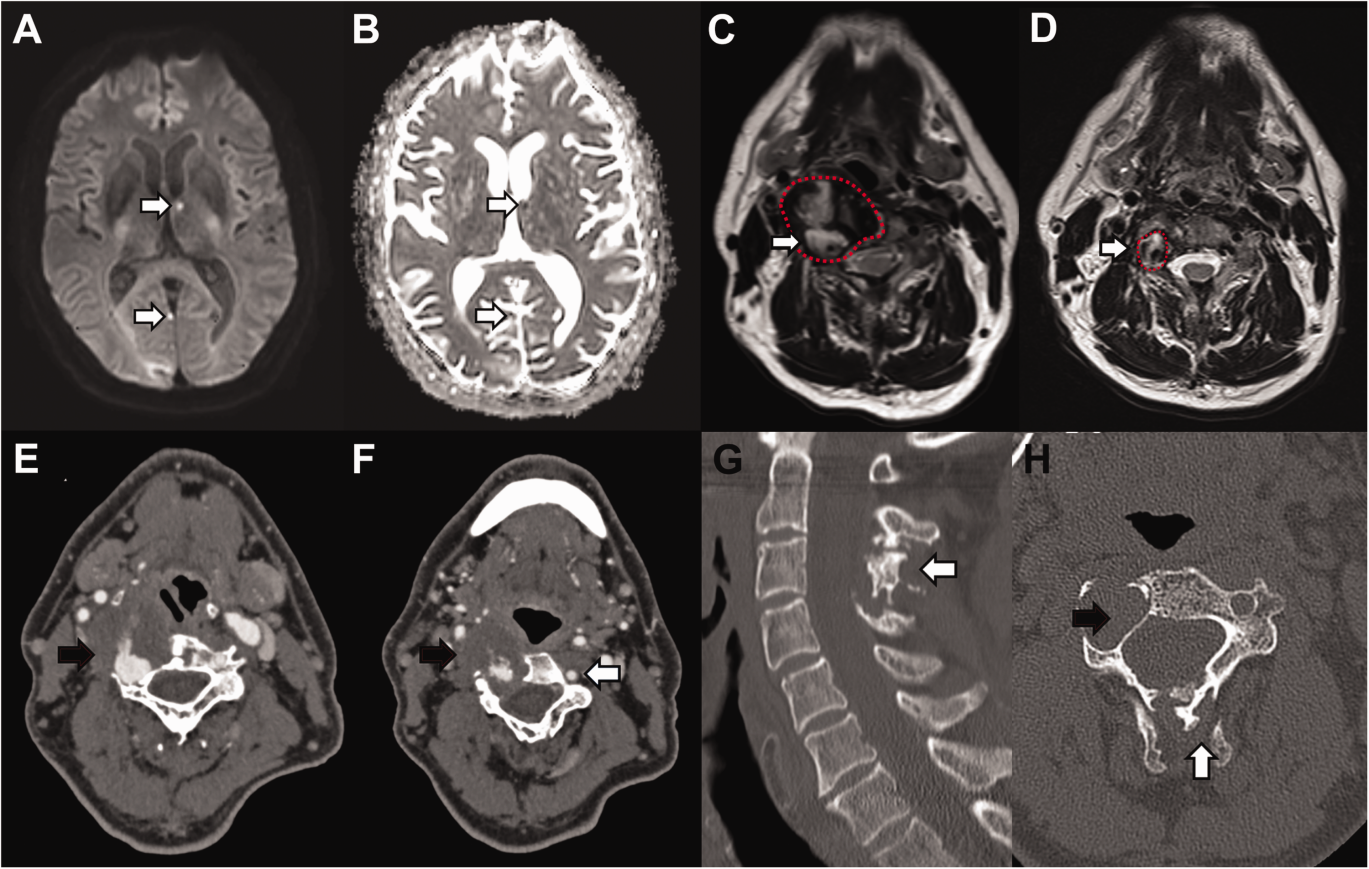
A and B: Multiple foci of restricted diffusion (white arrow), with corresponding low ADC value in the posterior circulation involving the right occipital lobe, left thalamus and cerebellum (not shown), in keeping with acute embolic infarction. Older non-restricting foci of embolic infarction were also evident on T2 sequences (not shown). C: T2 weighted sequence of the neck at the C4 vertebral level demonstrating an abnormal right vertebral artery (red broken line) with a central flow void and abnormal circumferential thick mixed T2 signal material (white arrow). Maximal diameter of the fusiform aneurysm was up to 35mm. D: T2 weighted sequence of the neck at the C4 vertebral level five years prior to presentation confirming significant growth (white arrow) of the right vertebral artery (red broken line). E and F: CT angiography demonstrated a grossly abnormal right vertebral artery measuring up to 5cm in diameter. The aneurysm demonstrated thick thrombus lining the lumen (black arrow) with areas of fissuring and crenulation. The contralateral left vertebral artery was of normal calibre and appearances (white arrow). G: Sagittal bone window reconstruction of CT demonstrates abnormal appearance to the spinous processes (white arrow) of C2 to C4 consistent with historic trauma. H: Axial CT at C3 level shows a grossly expanded right foramen transversarium consistent with a chronic process of fusiform aneurysm formation (black arrow).

Initial CT identified hypoattenuation in the right occipital lobe consistent with infarction subsequently confirmed on MR but with multiple further foci of infarction in the posterior circulation involving the right occipital lobe, left thalamus and cerebellum in keeping with acute embolic infarction (Figure 1(a) and (b)). The T2 sequences showed older non-restricting foci of embolic infarction in the posterior circulation with an abnormal vertebral artery (Figure 1(c)), measuring up to 35 mm in maximal diameter, which was significant dilated from previous imaging (Figure 1(d)).

CT angiography demonstrated a grossly dilated right vertebral artery measuring up to 35 mm in diameter isolated to the V2 (foraminal segment). Areas of stenosis were related to the constraint of the aneurysmal vertebral artery in the transverse foramina, which would predispose to turbulent flow and hence aneurysm progression and thrombus formation. The aneurysm had a thick thrombus lining the lumen with areas of fissuring and crenulation (Figure 1(e) and (f)), the aneurysm spanned over half the length of the extracranial segment. Given the history of possible significant neck injury, and evidence of historic bony injury (Figure 1(g)), we postulated this may have caused asymptomatic vertebral artery dissection with resultant chronic fusiform aneurysm formation. The aneurysm was associated with bony re-modelling (Figure 1(h)), of the right lateral cervical spine (C2-C5) implying considerable chronicity and exerting mass effect upon the adjacent pharynx.

**Figure 2.**
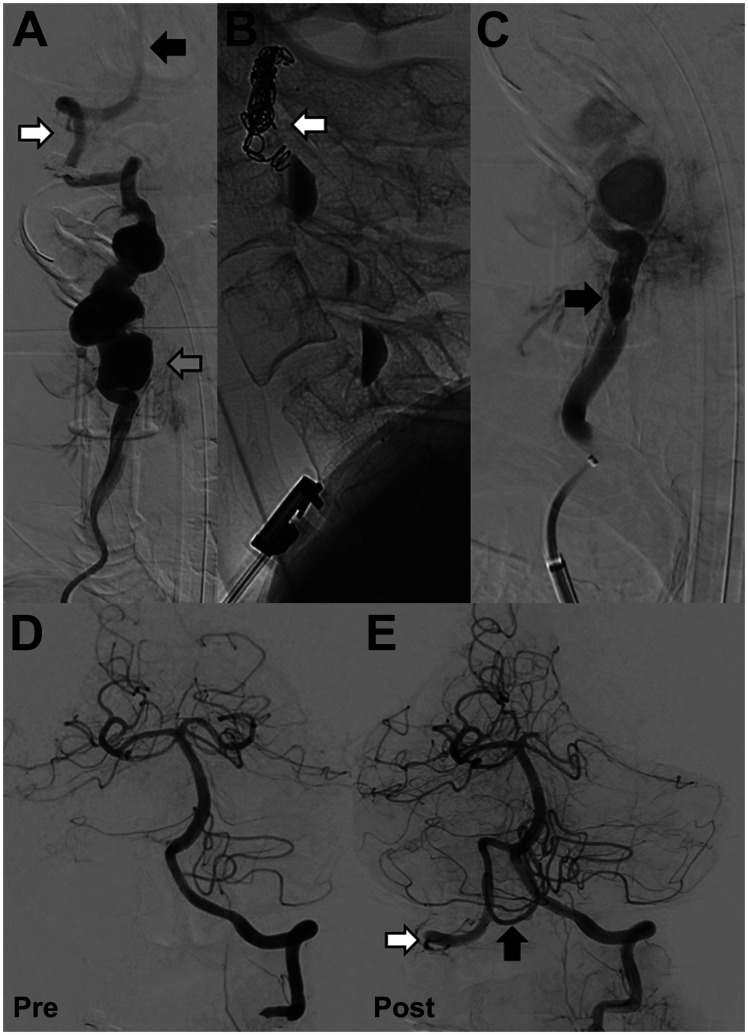
A: Digital subtraction angiography (DSA)(anterior-posterior view) confirmed a chronic fusiform aneurysmal right vertebral artery with areas of saccular dilatation (grey arrow). The distal V3 segment of the vertebral artery (white arrow) and intra-cranial basilar artery were normal (black arrow). B: DSA (lateral view) endovascular embolization was performed with distal micro-vascular plug and platinum coils (white arrow). Contrast stagnation can be seen in the areas of saccular dilatation. C: Proximal cervical vertebral artery embolization was performed with a single micro-vascular plug (black arrow). D: Pre-embolization angiogram with injection in the left vertebral artery demonstrates normal appearances with no opacification of the right posterior inferior cerebellar artery territory (PICA). E: Post-embolization retrograde flow of contrast from the left vertebral artery fills the intra-dural right vertebral artery (white arrow) and perfuses the right PICA (black arrow).

This case was urgently discussed in a dedicated neurovascular multi-disciplinary team meeting, composing of interventional neuro-radiologists, neuro-surgeons and neurologists. Careful discussion was undertaken with the patient regarding the risk benefit of best medical therapy, endovascular or open surgical treatment. Given the evidence of historic ‘silent’ thrombo-embolic events, based on established non-restricting multifocal elevated T2 foci on MRI, and the theoretical risk of future catastrophic major vessel thromboembolism in the posterior circulation the patients preference was for interventional treatment. Urgent endovascular evaluation and treatment of the aneurysmal right vertebral artery to prevent further embolic ischaemic strokes or rupture of the aneurysm was undertaken. Digital subtraction angiography (DSA) confirmed a chronic fusiform aneurysmal right vertebral artery with areas of saccular dilatation. The origin, V1 segment (pre-foraminal), V3 segment (atlantic extra-dural segment) and V4 segment (intra-cranial) and basilar artery were normal (Figure 2(a)).

## Procedure

The patient underwent general anaesthesia prior to endovascular intervention (American Society of Anaesthesiologists (ASA) grade 1). Procedure was performed in a biplane neuro-interventional suite (Allura Clarity FD20, Philips, Netherlands). Under ultrasound guidance the right common femoral artery was punctured and a 6 Fr sheath inserted. 5000units of intra-arterial heparin was administered. Initial angiogram in the left vertebral artery confirmed excellent calibre and supply to the basilar and posterior circulation. An unfolded aortic arch was encountered but catheterisation with a 5 Fr vertebral curve catheter of the right subclavian artery was relatively uncomplicated. An exchange manoeuvre was performed to gain stable access with a 80 cm 8 Fr Neuronmax (Penumbra Inc., Ca, USA) long sheath. A 5 Fr Sofia (Microvention, Ca, USA) with an Excelsior XT-27 microcatheter (Stryker, MI, USA) and Synchro microwire (Stryker, MI, USA) was navigated through to the V3 segment. Embolisation was performed with a proximal 5 mm micro-vascular plugs (MVP-3 Microvascular Plug)(Medtronic, Min, USA) and distal 3 mm plug and five platinum coils (Microplex) (Microvention, Ca, USA) (Figure 2(b) and (c)). Post-embolisation reflux of contrast from the left vertebral artery retrogradely perfused the intra-dural right vertebral artery with preserved perfusion of the right posterior inferior cerebellar artery (Figure 2(d) and (e)). Common femoral arterial puncture was closed with a Perclose ProGlide (Abbott, IL, USA).

The patient was transferred to the neuro-intensive care unit and recovery was unremarkable, subsequently he was discharged on dual anti-platelet therapy (aspirin and clopidogrel) for 21 days, then clopidogrel 75 mg once daily lifelong, as per current secondary prevention guidelines,^19^ atorvastatin 40 mg and amlodipine 5 mg once daily. Two months post procedure time of flight MR angiography confirmed no flow in the aneurysmal segment and standard imaging did not demonstrate any post-procedural infarction. The patient returned to his normal pre-procedural state leading an independent life with no new neurological deficit.

## Discussion and literature review

To date we have identified twenty-seven patients where a chronic or primary aneurysm of the extracranial circulation was treated,^5^,^7^,^9–15^,^17^,^20–29^ twelve of which were not associated with a connective tissue disorder (Table 1), acute traumatic aneurysms were not reviewed. Nine of these cases were treated with endovascular techniques^7,14,16,21,24–27,30^ whilst the remainder underwent surgical resection with additional reconstruction or bypass. These studies are significantly limited by both clinical and radiological follow up.

**Table 1.** Extracranial vertebral artery aneurysm with no known hereditary connective tissue disorder and unknown aetiology (excluding acute traumatic aneurysm and pseudoaneurysm).

References	Presentation	Treatment	Follow up
Laurian et al.^27^	57M	Detachable ballloon	Unknown
Thompson et al.^9^	73M. Non-tender pulsatile mass. Dysphagia, chest pain and dyspnoea.	Ligation and resection	Unknown
Clark et al.^28^	33M. Diplopia, dysarthria and vertigo. Military missile injury 12 years previous.	Resection and anastomosis.	Not reported
Rifkinson-Mann et al.^10^	31F. Firm non-tender pulsatile neck mass.	Ligation and resection	Not reported.
Ekeström et al.17	25F. Neck and right arm pain, left facial paraesthesia.	Ligation	Symptom free 4.5 years
Habozit and Battistelli^11^	40M. Neck pain.	Bypass and ligation.	Postoperative course uneventful
Buerger et al.^12^	62F. Painful pulsatile swelling.	Ligation	No post-operative complications
Suliman et al.^13^	50F. Painful posterior neck mass and headache.	Ligation and resection	Well at 4 years.
Gallot et al.^29^	42F. SAH. Basilar aneurysm embolised. Incidental VA aneurysm.	Bypass and ligation	1 month. Improving facial paresis.
Stavrinou et al.^14^	61F. 6 month neck pain and brachialgia.	Proximal endovascular occlusion.	Not reported
Shang et al.^16^	26M. Right arm and chest pain.	Coil embolization. Covered subclavian stent.	Symptom free at 5 months.
Kikuchi and Kowada^15^	14F. Neck pain and swelling.	Resection and venous graft.	C5 palsy at 2 years.

Although primary and chronic dissection extracranial vertebral artery aneurysms may well be etiologically heterogeneous the long term management and risk of future rupture or thromboembolic events could be considered similar. No large series of histological studies of chronic extracranial vertebral artery aneurysms is available but the few case reports are that such aneurysms do contain laminated thrombus and the media is fragmented and replaced by blood and hyaline.^9^,^12^ In addition analysis of dissecting intracranial vertebral artery aneurysm have shown specific features including; fragmentation of the internal elastic lamina, neoangiogenesis within the thickened intima, intramural haemorrhage and thrombus formation, and angiogenesis within the thrombus.^31^ Furthermore histological analysis of extracranial carotid aneurysms has shown that the both thrombus and calcification is present in both degenerative or chronic dissection associated aneurysms and that both types demonstrate elastin loss from the media wall.^32^ As such we might speculate that long term sequelae of chronic extracranial vertebral artery aneurysms is similar to that of extracranial internal carotid artery aneurysm where there is a stroke prevalence of 50% and a mortality of 60–70% when untreated.^33^

Historically surgical management of the extra-cranial vertebral artery lesions was challenging with a variety of approaches reported including vertebral endarterectomy with or without patch, resection of the vertebral artery lesion with an end-to-end anastomosis with or without vein graft,^14^ distal and proximal ligation of the aneurysm and then resection of the lesion,^9^ and proximal ligation of the vertebral artery with vein bypass.^10^ Surgical ligation of the vertebral artery was advocated as the treatment of choice, however significant complications due to haemodynamic changes in the brainstem, including death, were reported.^1^ The largest single centre report of extracranial vertebral artery aneurysm surgery in 9 patients reported post-operative transient ischaemic attacks, dizziness and headaches in 51.7%,^5^ however this may be considered an older surgical series before the advent of advanced and accessible endovascular treatments.

With the evolution in endovascular techniques proximal balloon occlusion of the vertebral artery was reported,^29^ as well as coil embolisation^16^ and stenting with additional endosaccular coils when treating intracranial dissection.^34^ Although intracranial vertebral artery dissection aneurysms are now often treated with intra-luminal stent placement these scenarios are entirely different as preservation of the vessel is required. In this reported case preoperative balloon occlusion test to assess sufficient perfusion from the contralateral vertebral artery was not deemed necessary as the left vertebral artery was clearly of excellent caliber as were the internal carotid arteries both on CT and catheter angiography. This decision avoided requirement of a second arterial access site and unnecessary balloon inflation in the diseased right vertebral artery, also navigating a device through the chronic aneurysm to the V3 segment would risk further thromboembolic event. Also this would avoid repeated catheterization of the healthy left vertebral artery, that is to say avoiding injury and jeopardizing being able to safely occlude the diseased right vertebral artery. However the authors do appreciate that some practitioners would only perform vessel sacrifice following balloon test occlusion.

Although direct comparison between surgery and endovascular treatment of extracranial vertebral artery aneurysm has not been performed or likely to be feasible, given the low incidence, treatment of intracranial vertebral artery dissection aneurysms has been subject to meta-analysis from which one might glean some information. Both surgical trapping and stent-assisted coiling demonstrated high rates of good long-term neurologic outcomes and low recurrence and mortality rates in intracranial dissecting vertebral artery aneurysms, but one should consider these in the context of acute dissection and the pre-existing morbid state.^35^ Thus surgical trapping in this reported case would be technically feasible however endovascular occlusion was felt to be lower morbidity given the naturally less invasive manner of treatment. The position of the aneurysm also clearly did not risk injury to the anterior spinal artery or posterior inferior cerebellar artery (PICA) which may have mandated either surgery or complex stent assisted treatment to preserve the vessel.

In the literature there were twenty-one patients with follow up which reported peri-operative complications in four patients and symptomatic improvement was reported in fourteen patients (66%), one patient had recurrent radiculopathy, one had new radiculopathy and one endovascular patient developed a delayed arterio-venous fistula. Therefore treatment of vertebral artery aneurysms can be deemed relatively safe and lead to improvement in local symptoms. The risk profile is similar to any endovascular vessel occlusion procedure.

Flow diverting stent placement was not favored in this case for several reasons. Primarily wall apposition plays an important role in speed of and likelihood of endothelialization of the flow diverting stent, this in turn is critically important for aneurysm occlusion.^36^ Technical factors would also preclude the use of flow diverting stent as the largest available measure 5 mm in maximal diameter, although the stent could be sized to the ‘normal’ proximal and distal vessel there would still be long length of stent with no apposition. The authors felt immediate vessel sacrifice with the microvascular plug device was the quickest option with the lowest risk profile. It was felt preservation of the distal extracranial vertebral artery (V3 segment) was not required as the main aim was to preserve the right PICA, which was clearly achieved by retrograde flow in the right V4 segment. Vessel sacrifice has long been known to be a definitive procedure to manage any aneurysm with low morbidity if adequate intracranial anastomosis and perfusion is confirmed. The microvascular plug (MVP) device has been primarily used in peripheral vascular procedures, such as in renal artery occlusion.^37^ The benefit of the MVP was ease of deployment and relative low cost compared to specific neurovascular devices, perhaps historical detachable balloon device would also have been a consideration,^21^ but have since been removed from the market. Complete aneurysm sac embolization was not undertaken as this was felt to be excessive as proximal and distal control was achieved and also this would allow some regression in the local mass effect given the distortion of the pharynx, in addition this would have required many coils with significant cost. Of note previous report of proximal ligation resulted in continued aneurysm filling from muscular anastomosis^20^ and a case treated with proximal endovascular embolisation resulted in persistent filling and even developed aberrant outflow to become fistulous.^7^ Therefore it is essential to obtain distal and proximal occlusion. In this case partial embolisation of the aneurysm sack was also performed to aid thrombosis and reduce the risk of recanalisation.

Rupture of an extracranial vertebral artery aneurysm can result in simple haematoma and neck pain^6^,^7^ however death from rupture into the thoracic cavity despite emergent endovascular balloon placement to control haemorrhage has been reported.^8^ The risk of lifetime rupture is unknown given the rarity of these lesions and the incidence is likely higher than reported as many cases remain asymptomatic. Most reports are associated with an underlying connective tissue disorder and growth of the aneurysm can be asymptomatic, aneurysms have been reported to measure up to 5 cm diameter, over ten times the normal calibre.^38^ Continued aneurysm growth must be taken into consideration, in a paediatric patient with Neurofibromatosis type-1 followed up from age one to seven showed progressive enlargement of a vertebral aneurysm with associated upper limb symptoms, as well as massive aneurysmal dilatation of the thoracic aorta.^39^ This highlights the importance of appropriately counselling the patient on treatment and possible future growth if untreated.

## Conclusion

Extracranial vertebral artery aneurysms are a rare entity and can present with posterior circulation ischaemic strokes. They are most often associated with an underlying connective tissue disorder; however, primary aneurysm formation or previous trauma are possibilities to consider. Endovascular treatment with vessel sacrifice is an effective treatment with low morbidity and we believe the MVP device to be a efficacious option in the vertebral artery.

## References

[1] Matas R. Traumatisms and traumatic aneurisms of the vertebral artery and their surgical treatment with the report of a cured case. Ann Surg 1893; 18: 477–521. PMC1493274 17859982

[2] Krueger BR Okazaki H. Vertebral-basilar distribution infarction following chiropractic cervical manipulation. Mayo Clin Proc 1980; 55: 322–332. 7374218

[3] Do KH Leggit JC Galifianakis A. Extracranial vertebral artery dissecting aneurysm with snowboarding: a case report. Curr Sports Med Rep 2018; 17: 16–19. 29315103 10.1249/JSR.0000000000000441

[4] Egnor MR Page LK David C. Vertebral artery aneurysm – a unique hazard of head B banging by heavy metal rockers. Pediatr Neurosurg 1991; 17: 135–138. 1819327 10.1159/000120583

[5] Schievink WI Piepgras DG. Cervical vertebral artery aneurysms and arteriovenous fistulae in neurofibromatosis type 1: case reports. Neurosurgery 1991; 29: 760–765. 1961409 10.1097/00006123-199111000-00020

[6] Morasch MD Phade SV Naughton P , et al. Primary extracranial vertebral artery aneurysms. Ann Vasc Surg 2013; 27: 418–423. 23540677 10.1016/j.avsg.2012.08.002

[7] Ushikoshi S Goto K Uda K , et al. Vertebral arteriovenous fistula that developed in the same place as a previous ruptured aneurysm: a case report. Surg Neurol 1999; 51: 168–173. 10029422 10.1016/s0090-3019(98)00011-1

[8] Miyazaki T Ohta F Daisu M , et al. Extracranial vertebral artery aneurysm ruptured into the thoracic cavity with neurofibromatosis type 1: case report. Neurosurgery 2004; 54: 1517–1520. 15157311 10.1227/01.neu.0000125547.31328.69

[9] Thompson JE Jr Eilber F Baker JD. Vertebral artery aneurysm: Case report and review of the literature. Surgery 1979; 85: 583–585. 432821

[10] Rifkinson-Mann S Laub J Haimov M. Atraumatic extracranial vertebral artery aneurysm: case report and review of the literature. J Vasc Surg 1986; 4: 288–293. 3528535

[11] Habozit B Battistelli JM. Spontaneous aneurysm of the extracranial vertebral artery associated with spinal osseous anomaly. Ann Vasc Surg 1990; 4: 600–603. 2261329 10.1016/S0890-5096(06)60847-9

[12] Buerger T Lippert H Meyer F , et al. Aneurysm of the vertebral artery near the atlas arch. J Cardiovasc Surg 1999; 40: 387–389. 10412926

[13] Suliman AEA Hamid HK Mekki SO. An unusual case of a giant extracranial vertebral artery aneurysm. Vascular 2019; 27: 427–429. 30975041 10.1177/1708538119843403

[14] Stavrinou LC Stranjalis G Stavrinou PC , et al. Extracranial vertebral artery aneurysm presenting as a chronic cervical mass lesion. Case Rep Med 2010; 2010: 1–3. 10.1155/2010/938219 PMC2850509 20379376

[15] Kikuchi K Kowada M. Nontraumatic extracranial aneurysm of the vertebral artery. Surg Neurol 1983; 19: 425–427. 6845154 10.1016/0090-3019(83)90139-8

[16] Shang EK Fairman RM Foley PJ , et al. Endovascular treatment of a symptomatic extracranial vertebral artery aneurysm. J Vasc Surg 2013; 58: 1391–1393. 23561429 10.1016/j.jvs.2013.01.040

[17] Ekeström S Bergdahl L Huttunen H. Extracranial carotid and vertebral artery aneurysms. Scand J Thorac Cardiovasc Surg 1983; 17: 135–139. 6612257 10.3109/14017438309109877

[18] Merwick A Werring D. Posterior circulation ischaemic stroke. Bmj 2014; 348: g3175–g3175. 24842277 10.1136/bmj.g3175

[19] Stroke and transient ischaemic attack in over 16s: diagnosis and initial management. NICE guideline, www.nice.org.uk/guidance/ng128 (2019, accessed 5 May 2021).

[20] Schubiger O Yasargil MG. Extracranial vertebral aneurysm with neurofibromatosis. Neuroradiology 1978; 15: 171–173. 97573 10.1007/BF00329063

[21] Negoro M Nakaya T Terashima K , et al. Extracranial vertebral artery aneurysm with neurofibromatosis. Endovascular treatment by detachable balloon. Neuroradiology 1990; 31: 533–536. 2112736 10.1007/BF00340136

[22] Ohkata N Ikota T Tashiro T , et al. A case of multiple extracranial vertebral artery aneurysms associated with neurofibromatosis. No Shinkei Geka 1994; 22: 637–641. 8078595

[23] Kim HS Choi CH Lee TH , et al. Fusiform aneurysm presenting with cervical radiculopathy in Ehlers-Danlos syndrome. J Korean Neurosurg Soc 2010; 48: 528–531. 21430980 10.3340/jkns.2010.48.6.528 PMC3053548

[24] Peyre M Ozanne A Bhangoo R , et al. Pseudotumoral presentation of a cervical extracranial vertebral artery aneurysm in neurofibromatosis type 1: case report. Neurosurgery 2007; 61: E658–E658. 17881942 10.1227/01.NEU.0000290919.47847.D7

[25] Hiramatsu H Negoro M Hayakawa M , et al. Extracranial vertebral artery aneurysm associated with neurofibromatosis type 1. A case report. Interv Neuroradiol 2007; 13: 90–93. 20566083 10.1177/15910199070130S112 PMC3345472

[26] Uneda A Suzuki K Okubo S , et al. Neurofibromatosis type 1-associated extracranial vertebral artery aneurysm complicated by vertebral arteriovenous fistula after rrupture: case report and literature review. World Neurosurg 2016; 96: 609.e13–609.e18. 10.1016/j.wneu.2016.09.036 27647034

[27] Laurian C Georges B Richard T , et al. Interet de l’abord de l’artere vertebral dans son segment extracranien. A propos d’un aneurysme de l’artere vertebral en C3. J Mal Vasc (Paris) 1980; 5: 149–150. 7462838

[28] Clark JB Six EG Early CB. Resection and anastomosis of a cervical vertebral artery aneurysm. Microsurgery 1984; 5: 127–129. 6493027 10.1002/micr.1920050306

[29] Gallot JC Thomas P Douvrin F , et al. Treatment of extracranial vertebral aneurysm associated with two intracranial aneurysms – a case report. EJVES Extra 2005; 10: 142–145.

[30] Detwiler K Godersky JC Gentry L. Pseudoaneurysm of the extracranial vertebral artery. Case report. J Neurosurg 1987; 67: 935–939. 3681435 10.3171/jns.1987.67.6.0935

[31] Nakatomi H Segawa H Kurata A , et al. Clinicopathological study of intracranial fusiform and dolichoectatic aneurysms: insight on the mechanism of growth. Stroke 2000; 31: 896–900. 10753995 10.1161/01.str.31.4.896

[32] Welleweerd JC Nelissen BG Koole D , et al. Histological analysis of extracranial carotid artery aneurysms. PLoS One 2015; 10: e0117915. 25635813 10.1371/journal.pone.0117915 PMC4312019

[33] Zwolak RM Whitehouse WM Jr Knake JE , et al. Atherosclerotic extracranial carotid artery aneurysms. J Vasc Surg 1984; 1: 415–422. 6481891

[34] Lylyk P Cohen JE Ceratto R , et al. Combined endovascular treatment of dissecting vertebral artery aneurysms by using stents and coils. J Neurosurg 2001; 94: 427–432. 11235947 10.3171/jns.2001.94.3.0427

[35] Guan J Li G Kong X , et al. Endovascular treatment for ruptured and unruptured vertebral artery dissecting aneurysms: a meta-analysis. J NeuroIntervent Surg 2017; 9: 558–563. 10.1136/neurintsurg-2016-012309 27220870

[36] Aquarius R de Korte A Smits D , et al. The importance of wall apposition in flow diverters. Neurosurgery 2019; 84: 804–810. 29659995 10.1093/neuros/nyy092

[37] Jardinet T Bonne L Oyen R , et al. Initial experience with the microvascular plug in selective renal artery embolization. Vasc Endovascular Surg 2020; 54: 240–246. 31928203 10.1177/1538574419897500

[38] Hoffmann KT Hosten N Liebig T , et al. Giant aneurysm of the vertebral artery in neurofibromatosis type 1: report of a case and review of the literature. Neuroradiology 1998; 40: 245–248. 9592796 10.1007/s002340050576

[39] Pentecost M Stanley P Takahashi M , et al. Aneurysms of the aorta and subclavian and vertebral arteries in neurofibromatosis. Am J Dis Child 1981; 135: 475–477. 6786089 10.1001/archpedi.1981.02130290071024

